# A prospective, longitudinal, observational cohort study examining how glaucoma affects quality of life and visually-related function over 4 years: design and methodology

**DOI:** 10.1186/s12886-015-0088-x

**Published:** 2015-08-01

**Authors:** Michael Waisbourd, Samantha Parker, Feyzahan Ekici, Patricia Martinez, Rachel Murphy, Katie Scully, Sheryl S. Wizov, Lisa A. Hark, George L. Spaeth

**Affiliations:** Glaucoma Research Center, Wills Eye Hospital, 840 Walnut Street, Philadelphia, PA 19107 USA

**Keywords:** Glaucoma, Vision-related quality of life, Performance-based measures

## Abstract

**Background:**

The aim of this study is to summarize the design and methodology of a prospective, longitudinal, observational cohort study to investigate how glaucoma affects patients’ quality of life and visually-related function over a 4-year period.

**Methods/Design:**

One hundred sixty-one (161) subjects were enrolled in this ongoing study. Patients between the ages of 21–85 years with a minimum 2-year diagnosis of primary open-angle glaucoma, chronic primary angle-closure glaucoma or pseudoexfoliation glaucoma were included. Each patient visited Wills Eye Hospital for a baseline visit. Follow-up is planned for a minimum of 4 years, with annual visits. Each visit includes (1) Clinical evaluation: a slit lamp examination, fundoscopy, intraocular pressure measurement, visual field examination, spectral domain optical coherence tomography, Pelli-Robson Contrast Sensitivity test and the Spaeth-Richman Contrast Sensitivity test; (2) a performance based measure: the Compressed Assessment of Ability Related to Vision; and (3) Subjective measures of vision-related quality of life (the National Eye Institute Visual Functioning Questionnaire 25 and the Modified Glaucoma Symptom Scale).

**Discussion:**

The results of this ongoing, prospective, longitudinal study are expected to shed light on the relationships between clinical measures, performance-based measures and subjective measures of well-being, in order to assess changes in the quality of life and the ability to function of patients with glaucoma over time.

## Background

Glaucoma is a chronic neuro-degenerative disorder of the optic nerve in which death of the retinal ganglion cells and loss of optic nerve axons result in structural and functional deficits. Glaucoma is the leading cause of irreversible blindness worldwide and a major public health concern. Using the United Nations’ population projections, it is estimated that over 79 million people will be diagnosed with glaucoma by the year 2020 and over 111.8 million by 2040 [1, 2]. Glaucoma, initially asymptomatic, is a major cause of severe vision impairment. Advanced glaucoma can significantly degrade a patient’s vision-related quality of life (VRQoL) [3].

Although widely accepted as an important component of patient health, quality of life (QoL) is seldom assessed in daily clinical practice. This is partially due to limitations of validated VRQoL instruments. Generic instruments that assess overall QoL often underestimate the effects of specific domains, such as vision. Highly specific instruments-such as glaucoma patient questionnaires-underestimate generic aspects, such as anxiety [4]. Addressing patients’ VRQoL requires the use of scientifically valid and reliable instruments. Even less frequently assessed is patients’ ability to function in daily life, despite this being, along with a QoL, their major concern. This study aims to link subjective assessments of one’s QoL with objective measures of one’s function and glaucoma severity in order to properly assess and address VRQoL.

There are three distinct approaches to measuring the impact of glaucoma on individuals’ lives: 1) clinical measures (for example, visual acuity (VA), contrast sensitivity (CS), and visual field (VF)), 2) self-reported measurements of subjective well-being (QoL), and 3) performance-based assessments of the ability to carry out daily activities [5, 6]. Many studies have investigated the QoL of patients with glaucoma [7–15]. However, few have combined all three approaches, including performance-based measures and even fewer have utilized longitudinal designs [16–19]. One of the major strengths of the current study is that the longitudinal design will allow for a comprehensive assessment of QoL of patients with glaucoma over time.

This manuscript describes the design and methodology of an ongoing, prospective, longitudinal, observational cohort study, which aims to investigate how glaucoma affects patients’ QoL and visually related functions over a 4-year period.

## Methods/Design

### Participants

The Institutional Review Board at Wills Eye Hospital approved the study procedure, which was conducted in accordance with the Declaration of Helsinki. Informed consent was obtained from all participants. The consent was in accordance with Health Insurance Portability and Accountability Act (HIPAA) regulations.

Investigators in the Glaucoma Research Center reviewed the electronic medical records of patients being cared for by the Glaucoma Service of the Wills Eye Hospital to identify eligible patients.

One hundred sixty-one (161) patients are enrolled in the study. Patient eligibility is determined according to the inclusion and exclusion criteria specified in Table 1.

**Table 1:** Inclusion and exclusion criteria for patient enrollment

Inclusion criteria	
• Minimum 2-year diagnosis of primary open-angle glaucoma, primary angle-closure glaucoma or pseudoexfoliation glaucoma	
• Disc Damage Likelihood Scale stages 5 through 8 in at least one eye with characteristic visual field loss	
• Age between 21 and 85 years	
• Able to understand and speak English	
Exclusion criteria	
• Unlikely to be available for annual ocular examination and reassessment across a 4-year period	
• Neurological or musculoskeletal diseases, including dementia that would influence performance on activities of daily living	
• Incisional eye surgery within the past 3 months	
• Laser therapy within the previous month	
• Any cause for visual impairment other than glaucoma	
• Any medical condition which in the investigator’s opinion would preclude the patient from providing reliable and valid data (e.g., cognitive impairment)	

Patients with moderate-stage glaucoma without any other eye diseases are included. Glaucoma is considered present if the patient has glaucomatous optic neuropathy and characteristic VF loss in at least one eye. The Disc Damage Likelihood Scale (DDLS) is used to evaluate the extent of optic disc damage caused by glaucoma. The DDLS generates a score from 1–10 based on the rim/disc ratio (rather than cup/disc ratio) and the size of the optic nerve [20–24]. Patients presenting with DDLS stages 5 through 8 are eligible to participate in the study. Patients with DDLS stages 1 through 4 often do not exhibit VF loss due to glaucoma. Patients with DDLS 9 and 10 have extensive disc and field damage, making deterioration of the optic rim difficult to detect and establish [24]. Due to these reasons, stages 1 through 4 and 9 through 10 have been excluded from the study.

To maximize enrollment and minimize attrition, participants were provided with consistent encouragement from the Wills Eye Glaucoma ophthalmologists to participate in this clinical research project. The longitudinal nature of the project was thoroughly emphasized to patients during the consenting process: patients agreed to participate for the entire 4-year period of the study. Personal letters continue to be mailed (printed in large font) to patients in order to maintain positive, personalized contact. Additionally, the researchers call patients two days before a scheduled follow-up appointment as a reminder. Compensation is provided for the completion of each of the five annual assessments.

### Research instruments

An ocular examination and evaluation of both VRQoL and performance-based visual functioning is performed at each visit.

The ocular examination consists of best-corrected VA, measurement of intraocular pressure (IOP), a slit lamp examination of the anterior segment and a fundus examination. Patients’ current symptoms, health problems, medications, and ocular co-morbidities are also documented. VAs (monocular and binocular) are scored by counting how many letters can be read correctly using criteria from the Lighthouse Early Treatment Diabetic Retinopathy Study (ETDRS) charts. The score is then converted to a logarithm of the minimum angle of resolution (LogMAR). IOPs are measured using a calibrated Goldmann applanation tonometer. The Humphrey 24–2 Swedish Interactive Threshold Algorithm (SITA) Standard perimeter (Zeiss Meditec, Dublin, CA) is used to test VF. A Cirrus optical coherence tomography (OCT) (Zeiss Meditec, Dublin, CA) instrument obtains optic-nerve head imaging for each patient.

The Pelli-Robson (PR) test and the Spaeth-Richman Contrast Sensitivity (SPARCS) test are used to measure contrast sensitivity. Contrast threshold is a measure of the ability of the visual system to distinguish the brightness of an object against a background. The PR is a contrast sensitivity test with 8 lines of horizontal capital letters measuring central vision. Each set of three letters on the chart becomes progressively lower in contrast relative to the chart background. The patients are instructed to read as far down as they can see. The chart is mounted on a white wall with the patient sitting 1 meter in distance from the chart; the luminance of the test is at 85 candelas/m^2^ (cd/m^2^), with the accepted range being between 60 to 120 cd/m^2^ [25].

**Fig. 1:**
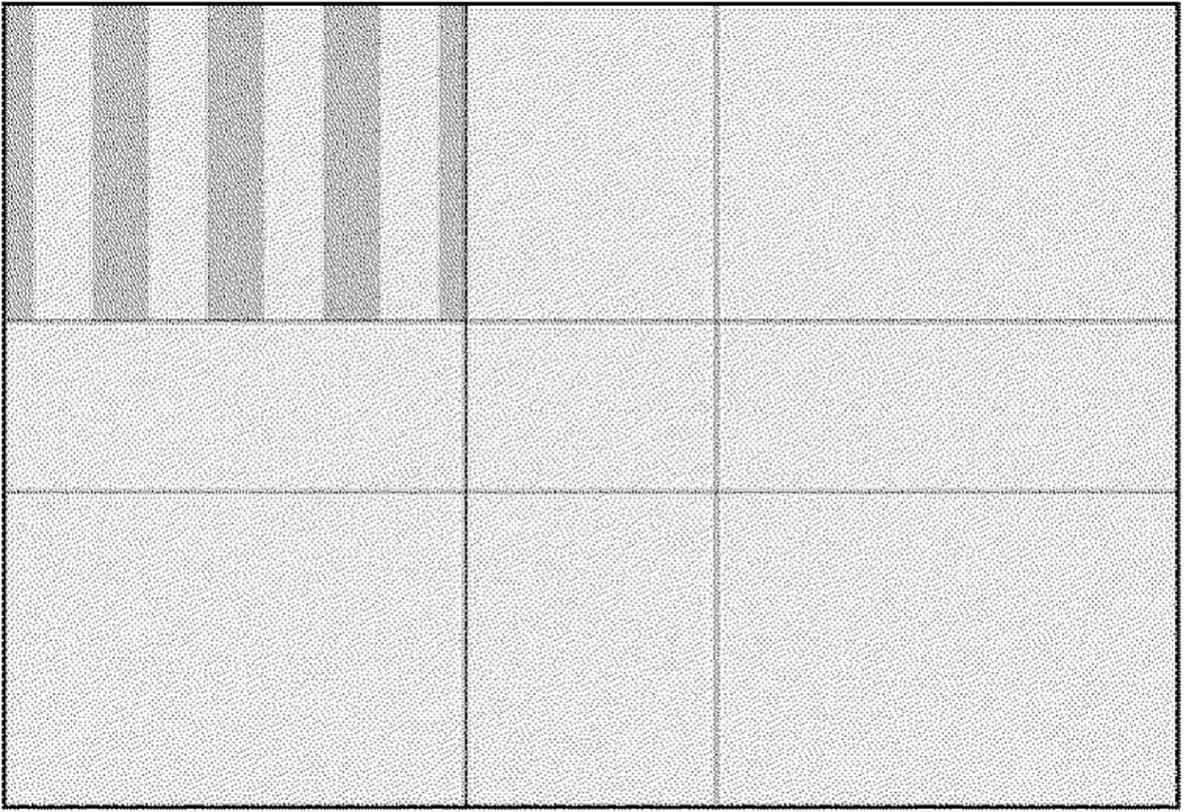
Spaeth Richman Contrast Sensitivity Test (SPARCS). Two parallel horizontal lines and 2 parallel vertical lines intersect to create 9 boxes, including a 5 cm rectangle in the center. Note the darkened square waves in the right upper quadrant. During a “set”, darkened square waves randomly appear briefly in 1 of the 5 tested areas while the other 4 areas remain the same shade as the background. Contrast between the bars and background decreases by 50 % after each cycle

The SPARCS test is a new method of measuring contrast sensitivity. It is performed on any standard computer with Internet access [26]. In this study, the SPARCS test was administered on the same computer, with standardized lighting conditions. SPARCS tests contrast sensitivity in 5 different areas of the VF: centrally and in 4 peripheral quadrants (Fig. 1). It does not require the patient to recognize objects or letters, which reduces the confounding effects of literacy, culture, and intelligence as well as the effects of macular function and VA. Correct and incorrect responses are recorded until the contrast threshold is determined in each of the 5 testing areas.

ADREV (assessment of disability related to vision) is a performance-based measure of vision related disability [16]. There are 9 items included in ADREV: 1) reading in reduced illumination 2) recognizing facial expression 3) detecting motion 4) reading signs at a distance 5) finding large and small objects spread around a room 6) navigating an obstacle course 7) putting a stick into holes of different sizes 8) telephone simulation and 9) matching socks. ADREV is the parent test for a newly modified performance-based assessment utilizing four of the original nine items. This instrument, the Compressed Assessment of Ability Related to Vision (CAARV), correlates excellently with the 9-item ADREV while only requiring between 10 and 15 min to complete (Table 2) [27]. The 4 CAARV items include: 1) computerized motion detection 2) recognizing facial expressions 3) reading street signs and 4) finding objects in a room. Patients complete CAARV sub-tests with both eyes open. They use their own appropriate refractive correction in order to simulate normal function. The patients complete two sub-tests in ambient lighting and two sub-tests in a dark room. Each item is scored from 0 to 7, 7 being perfect performance. The scores are then calculated for a total CAARV score as an aggregate of the scores of all four sub-tests.

**Table 2:** Compressed Assessment of Ability Related to Vision (CAARV)

1. Computerized motion detection
A large black cross against a white background on a computer screen provides a point of fixation. While fixating on the cross, one at a time, 14 balls of different sizes and colors move diagonally across the screen from either the right or the left side at a constant speed. Yellow, red, or blue balls are used. The patient is asked to count the number of moving balls. Each ball seen counts as ½ point. Highest score is 7 and lowest score is 0.
2. Facial expression recognition
Seven full-face professional, colored photos of varying sizes and facial expressions (angry, sad, happy, or surprised) are presented on a computer screen at a distance of ½ meter. The patient receives one point for recognizing the facial expression. Score ranged from 0 to 7 with 7 being the highest score.
3. Recognizing street signs
Seven written word signs ranging from large to small are read at a distance of 4 meters. One character in each sign was changed from familiar phrases making the word difficult to guess. For example, the top sign reads SUGAR DANE, which is similar to the more familiar sugar cane. The patient is instructed not to guess. One point is given for each sign read correctly. Highest score is 7 and lowest score is 0.
4. Locating objects
Fourteen red and beige boxes of different sizes are scattered around the testing room (4 x 2 meters). Sample boxes are shown before test started. The patient attempts to locate the boxes while seated. Each box found is worth ½ point. Highest score is 7 and lowest 0.

The NEI-VFQ-25 includes a series of 25 questions pertaining to vision or feelings about a vision condition (Table 3). The patients select answers among a multiple-choice list of possible responses. The values of these answers are re-coded and converted to a 0 to 100 scale so that the lowest and highest possible scores are set at 0 and 100. Researchers average together the items within each subscale to create 12 subscale scores and can calculate an overall composite score by averaging the subscale scores, excluding the general-health-rating question [28].

**Table 3:** National Eye Institute Visual Functioning Questionnaire – 25 (NEI-VFQ-25)

1. In general, would you say that your overall health is:	
1) Excellent 2) Very Good 3) Good 4) Fair 5) Poor	
2. At the present time, would you say your eyesight using both eyes (with glasses or contact lenses, if you wear them) is:	
1) Excellent 2) Good 3) Fair 4) Very Poor 5) Completely Blind	
3. How much of the time do you worry about your eyesight?	
1) None of the time 2) A little of the time 3) Some of the time 4) Most of the time 5) All of the time	
4. How much pain or discomfort have you had in and around your eyes (for example, burning, itching, or aching)? Would you say it is:	
1) None 2) Mild 3) Moderate 4) Severe 5) Very severe	
PART 2 – Difficulty with Activities	
The next questions are about how much difficulty you have doing a certain activity, for each question answer: 1) No difficulty at all, 2) A little difficulty, 3) Moderate difficulty, 4) Extreme difficulty, 5) Stopped doing this because of eyesight, 6) Stopped doing this for other reasons or not interested in doing this.	
5. How much difficulty do you have reading ordinary print in newspapers?	
6. How much difficulty do you have doing work or hobbies that require you to see well up close, such as cooking, sewing, fixing things around the house, or using hand tools?	
7. Because of your eyesight, how much difficulty do you have finding something on a crowded shelf?	
8. How much difficulty do you have reading street signs or the names of stores?	
9. Because of your eyesight, how much difficulty do you have going down steps, stairs, or curbs in dim light or at night?	
10. Because of your eyesight, how much difficulty do you have noticing objects off to the side while you are walking along?	
11. Because of your eyesight, how much difficulty do you have seeing how people react to things you say?	
12. Because of your eyesight, how much difficulty do you have picking out and matching your own clothes?	
13. Because of your eyesight, how much difficulty do you have visiting with people in their homes, at parties, or in restaurants?	
14. Because of your eyesight, how much difficulty do you have going out to see movies, plays, or sports events?	
15. Now, I’d like to ask you about driving a car. Are you currently driving, at least once in a while?	
1) Yes 2) No	
15a. IF NO: Have you never driven a car or have you given up driving?	
1) Never drove 2) Gave up	
15b. IF GAVE UP DRIVING: Was that	
1) mainly because of your eyesight, 2) mainly for some other reason, 3) both your eyesight and other reasons?	
15c. IF CURRENTLY DRIVING: How much difficulty do you have driving during the daytime in familiar places?	
16. How much difficulty do you have driving at night?	
16a. How much difficulty do you have driving in difficult conditions, such as in bad weather, during rush hour, on the freeway, or in city traffic?	
PART 3 – Responses to Vision Problems	
The next questions are about how things you do may be affected by your vision. For each one, I’d like you to tell me if this is true for you 1) all, 2) most, 3) some, 4) a little, or 5) none of the time.	
17. Do you accomplish less than you would like because of your vision?	
18. Are you limited in how long you can work or do other activities because of your vision?	
19. How much does pain or discomfort in or around your eyes, for example, burning, itching or aching, keep you from doing what you’d like to be doing?	
For each of the following statements, please tell me if it is 1) definitely true, 2) mostly true, 3) mostly false, or 4) definitely false for you or you are 5) not sure.	
20. I stay home most of the time because of my eyesight.	
21. I feel frustrated a lot of the time because of my eyesight.	
22. I have much less control over what I do, because of my eyesight.	
23. Because of my eyesight, I have to rely too much on what other people tell me.	
24. I worry about doing things that will embarrass myself or others, because of my eyesight.	
25. I need a lot of help from others because of my eyesight.	

The study also uses a modified glaucoma symptom scale (MGSS), which includes 10 ocular complaints often associated with glaucoma or treatment for glaucoma (Table 4) [29]. For each eye, an initial four-level score is generated, with one signifying a very bothersome problem and four signifying the absence of a problem. This score is converted to a 0 to 100 scale, 0 signifying the presence of a very bothersome problem and 100 signifying the absence of a problem [29]. The final MGSS score is an unweighted average of the responses to all 10 items, averaged between the two eyes.

**Table 4:** Modified glaucoma symptom scale

Have you experienced any of the following problems in the last 4 weeks?			
(Please respond for both the left and right eye.)			
a. Burning, Smarting, Stinging			
Left Eye		Right Eye	
☐ Yes	How bothersome has it been?	☐ Yes	How bothersome has it been?
	______ Very		______ Very
	______ Somewhat		______ Somewhat
	______ A Little		______ A Little
☐ No (Not at all bothersome)		☐ No (Not at all bothersome)	
b. Tearing			
c. Dryness			
d. Itching			
e. Soreness, Tiredness			
f. Blurry/Dim Vision			
g. Feeling of Something in Your Eye			
h. Hard to See in Daylight			
i. Hard to See in Dark Place			
j. Halos Around Lights			

Baseline measurements were collected at the time of patient enrollment. At baseline, two VF examinations were collected and averaged to produce a baseline value. The patients enrolled in this study had minimum 2-year diagnosis of glaucoma, therefore all study patients had already underwent at least two VF examinations prior to their baseline assessment. At the 48-month follow up, two VFs will be conducted and compared to the baseline values. A complete ocular examination was conducted, in addition to CAARV, NEI-VFQ-25, Cirrus OCT, SPARCS, PR Contrast Sensitivity, and the MGSS on patients at the baseline visit and will be repeated for follow up visits at 12-, 24-, 36-, and 48-months.

### Statistical analysis and sample size justification

*Primary Outcome Variables* will be divided into three groups: 1) QoL (NEI-VFQ-25 and MGSS) 2) performance-based measures of visual function (CAARV) and 3) Clinical measures of vision (better and worse eye VF mean defect, better and worse eye VA, and binocular SPARCS contrast sensitivity). *Primary Exposure/Demographic Variables* will be characterized as baseline DDLS score, age, ethnicity, gender, type of glaucoma, and number of medical comorbidities. *Secondary Variables* will be characterized as better and worse eye IOP, treatments, and duration of treatment.

All primary and secondary variables measured at baseline will be compared using means, medians, standard deviations, ranges or frequencies, and percentages. At baseline, Spearman correlation coefficients will be provided for all pairs of outcome variables and demographic variables.

In order to characterize the longitudinal trajectory of VRQoL, clinical measures of vision, and vision-related functioning over a 4-year period, analysis of these outcomes will proceed in two stages. First, mixed effects linear regression will be used to model the average trajectory of the sample. This will identify the appropriate parametric forms for each outcome trajectory. The study collects outcomes at five different timepoints over the 4-year study period. Linear and quadratic curves will be calculated, as well as a saturated model that treats time as a categorical explanatory variable. This allows for estimation of the mean outcome score for each visit. The mixed effects model will account for correlation among repeated measurements from the same patient. Our goal in these analyses is to identify the most parsimonious model that adequately represents the shape of the outcome trajectory over time. Ideally, a linear model will be sufficient. It is possible, however, to imagine scenarios where a quadratic term may be required (e.g., initial decline followed by stabilization).

Second, because glaucoma is a disease with much subject heterogeneity, there are likely to be patients that differ from others in their experienced trajectories. MPlus software will be used to implement Growth Curve Mixture Modeling (GCMM) [30, 31]. This will group patients into a finite number of latent classes characterized by differing outcome trajectories over time. The model contains two parts: 1) the outcome model (i.e., the shape of the trajectory within each class), which is a mixed effects linear regression model and 2) the class probability model, which links the probability of being in any class with baseline exposure and demographic variables through a logistic regression model. Results from the initial mixed effects linear regression analysis will guide selection of the trajectory shapes for the GCMM. Results from the logistic regression portion of the model will allow for identification of baseline characteristics associated with differing changes in outcomes over time. Models will be fit with up to five latent classes using the Bayesian Information Criterion (BIC) to select the best-fitting model [32].

In order to determine whether changes in VRQoL are associated with changes in clinical measures of vision and performance-based measures of visual function, an extension of GCMM will be used to model to simultaneously model multiple outcome trajectories and identify classes based on the joint consideration of multiple outcomes. This will jointly model the trajectories of the primary outcome variables [32]. The resulting classes will have differing trajectory shapes for each outcome. Based on these results, we will be able to characterize the ways in which these outcomes change concurrently over time and the baseline patient characteristics that are associated with differing trajectories.

No sample size formulae exist for GCMM. A sufficient sample size of 160 patients was chosen in order to identify subgroups using the latent class analysis. The primary goal of this analysis is to estimate parameters of the trajectory in each class with appropriate precision. The precision of the confidence interval will be calculated for the class- and outcome-specific slopes, assuming that a linear model is sufficient to describe the trajectory of a particular outcome. Class sizes will vary based on outcome. For illustration, we estimated precision for class sizes of 20, 60, and 100. Each patient will have up to five observations. Assuming 5 % dropout per year, the average number of observations per patient is 4.5.

Table 5 shows the width of the confidence interval for the slopes for NEI-VFQ-25 Total Score and LogMAR VA. For reference, a slope of one would indicate a 12-unit change in the NEI-VFQ over 12 months. A slope of 0.01 for LogMAR would indicate a change of 0.12 units over 12 months. The calculations given in Table 5 assume an intra-subject correlation of 0.2. Higher values within subject correlation would result in less precision while lower correlation would result in increased precision.

**Table 5:** Sample confidence intervals for slopes of NEI-VFQ-25 and visual acuity

Number of subjects per class	Effective Sample Size N*4.5/(1 + (4.5-1)*0.2)	Distance from slope estimate to the two-sided 95 % confidence limits for the class-specific slope	
		NEI-VFQ (Std. Dev = 17)	LogMAR VA (Std. Dev = 0.2)
20	53	+/− 0.28	+/− 0.0033
60	159	+/− 0.16	+/− 0.0019
100	265	+/− 0.12	+/− 0.0014

*NEI-VFQ-25* National eye instate vision function questionnaire; LogMAR VA

### Missing data

Based on prior studies conducted in the Wills Eye Glaucoma Research Center, we anticipated losing approximately 5 % of enrolled patients per year (total 20 % across 4 years) due to attrition. Both mixed effects models and the GCMMs include all available data from all patients under the assumption that any missing data are missing at random. This is the approach researchers will take for the study’s primary analysis. As a sensitivity analysis, researchers will fit the final models using only patients with data from all visits.

## Discussion

“How can I improve, or at least maintain, the overall health and well being of my patients?” – is one of the most important questions every ophthalmologist should ask himself or herself, especially when treating patients with glaucoma.

Many visually impaired individuals have difficulty performing daily activities, such as reading, writing, eating, dressing and traveling from place to place. These difficulties tend to be even more pronounced in elderly patients, who may also have cognitive decline or other physical ailments in addition to their visual impairment. Understanding the impact of these difficulties on an individual’s QoL and how they are able to function in their daily lives is important and may have direct implication on clinical decision-making.

VRQoL is an elusive term, the meaning of which is it may vary widely between clinicians and their patients. Two patients with the same degree of visual impairment may have very different views on their VRQoL. In order to understand the impact of visual impairment on the ability of individuals to function, performance-based measures have been developed [16–18, 33–39]. The advantage of performance-based measures is that they provide an objective measurement of a patient’s abilities using standardized criteria. Ophthalmologists rarely assess patients’ performance-based functional ability, assuming that surrogates such as subjective self-report on well-being, VF, and VA are appropriate proxies [19, 40]. However, these proxies do not necessarily correlate with performance-based measures; further, some may be more important or relevant than others. Richman et al. investigated the relationships among three methods of assessing visual loss caused by glaucoma: (1) standard clinical tests of vision, (2) self-reported QoL, and (3) the ability to perform activities of daily living, which was assessed by ADREV. They concluded that performance-based testing and QoL evaluations were both independently important measures of health, and although related, were by no means the same [18].

The current study aims to investigate the complex relationships between between clinical measures (e.g., VA, IOP, VF, OCT and CS tests), performance-based measures (assessed by the CAARV), and subjective self-reported measures (NEI-VFQ-25 and MGSS). One of the major strengths of this study is investigation of change in these relationships over a 4-year period. The patients included in this study, diagnosed with moderate-stage glaucoma, are more likely to show progression over 4 years, compared to patients with earlier stage of the disease who were excluded from this study. We also excluded patients with advanced disease, who have already shown significant disease progression.

Previous studies have highlighted the importance of binocular visual acuity and contrast sensitivity as predictors of a patient’s ability to perform activities of daily living [17].

In the current study, we used the SPARCS test to measure contrast sensitivity, in addition to the PR test. The SPARCS, a novel computerized-based CS test, is able to differentiate between patients with glaucoma in comparison to normal controls, and may have the potential of becoming a useful surrogate for performance-based measures [26].

In summary, the results of this ongoing, prospective, longitudinal, observational cohort study are expected to provide useful information on how patients with glaucoma feel and function over a 4-year period. It is likely to identify the rate of change in VRQoL and ability to function compared with disease progression over time. The results of this study may allow clinicians to understand better how clinical measures reflect objective and subjective measures of QoL in this patient population.

## References

[1] Quigley HA, Broman AT (2006). The number of people with glaucoma worldwide in 2010 and 2020. Br J Ophthalmol.

[2] Tham YC, Li X, Wong TY, Quigley HA, Aung T, Cheng CY (2014). Global prevalence of glaucoma and projections of glaucoma burden through 2040: a systematic review and meta-analysis. Ophthalmology.

[3] Gutierrez P, Wilson MR, Johnson C, Gordon M, Cioffi GA, Ritch R, Sherwood M, Meng K, Mangione CM (1997). Influence of glaucomatous visual field loss on health-related quality of life. Arch Ophthalmol.

[4] Hickey A, Barker M, McGee H, O'Boyle C (2005). Measuring health-related quality of life in older patient populations: a review of current approaches. Pharmacoeconomics.

[5] Magacho L, Lima FE, Nery AC, Sagawa A, Magacho B, Avila MP (2004). Quality of life in glaucoma patients: regression analysis and correlation with possible modifiers. Ophthalmic Epidemiol.

[6] Spaeth G, Walt J, Keener J (2006). Evaluation of quality of life for patients with glaucoma. Am J Ophthalmol.

[7] Gracitelli CP, Abe RY, Tatham AJ, Rosen PN, Zangwill LM, Boer ER, Weinreb RN, Medeiros FA (2015). Association between progressive retinal nerve fiber layer loss and longitudinal change in quality of life in glaucoma. JAMA Ophthalmol.

[8] van Gestel A, Webers CA, Beckers HJ, van Dongen MC, Severens JL, Hendrikse F, Schouten JS (2010). The relationship between visual field loss in glaucoma and health-related quality-of-life. Eye (Lond).

[9] McKean-Cowdin R, Wang Y, Wu J, Azen SP, Varma R, Los Angeles Latino Eye Study Group (2008). Impact of visual field loss on health-related quality of life in glaucoma: the Los Angeles Latino Eye Study. Ophthalmology.

[10] Freeman EE, Munoz B, West SK, Jampel HD, Friedman DS (2008). Glaucoma and quality of life: the Salisbury Eye Evaluation. Ophthalmology.

[11] Hyman LG, Komaroff E, Heijl A, Bengtsson B, Leske MC, Early Manifest Glaucoma Trial Group (2005). Treatment and vision-related quality of life in the early manifest glaucoma trial. Ophthalmology.

[12] Jampel HD, Schwartz A, Pollack I, Abrams D, Weiss H, Miller R (2002). Glaucoma patients' assessment of their visual function and quality of life. J Glaucoma.

[13] Janz NK, Wren PA, Lichter PR, Musch DC, Gillespie BW, Guire KE, Mills RP, Group CS (2001). The Collaborative Initial Glaucoma Treatment Study: interim quality of life findings after initial medical or surgical treatment of glaucoma. Ophthalmology.

[14] Janz NK, Wren PA, Lichter PR, Musch DC, Gillespie BW, Guire KE (2001). Quality of life in newly diagnosed glaucoma patients : The Collaborative Initial Glaucoma Treatment Study. Ophthalmology.

[15] Hochberg C, Maul E, Chan ES, Van Landingham S, Ferrucci L, Friedman DS, Ramulu PY (2012). Association of vision loss in glaucoma and age-related macular degeneration with IADL disability. Invest Ophthalmol Vis Sci.

[16] Lorenzana L, Lankaranian D, Dugar J, Mayer J, Palejwala N, Kulkarni K, Warrian K, Boghara Z, Richman J, Wizov S (2009). A new method of assessing ability to perform activities of daily living: design, methods and baseline data. Ophthalmic Epidemiol.

[17] Richman J, Lorenzana LL, Lankaranian D, Dugar J, Mayer J, Wizov SS, Spaeth GL (2010). Importance of visual acuity and contrast sensitivity in patients with glaucoma. Arch Ophthalmol.

[18] Richman J, Lorenzana LL, Lankaranian D, Dugar J, Mayer JR, Wizov SS, Spaeth GL (2010). Relationships in glaucoma patients between standard vision tests, quality of life, and ability to perform daily activities. Ophthalmic Epidemiol.

[19] Wei H, Sawchyn AK, Myers JS, Katz LJ, Moster MR, Wizov SS, Steele M, Lo D, Spaeth GL (2012). A clinical method to assess the effect of visual loss on the ability to perform activities of daily living. Br J Ophthalmol.

[20] Bayer A, Harasymowycz P, Henderer JD, Steinmann WG, Spaeth GL (2002). Validity of a new disk grading scale for estimating glaucomatous damage: correlation with visual field damage. Am J Ophthalmol.

[21] Danesh-Meyer HV, Ku JY, Papchenko TL, Jayasundera T, Hsiang JC, Gamble GD (2006). Regional correlation of structure and function in glaucoma, using the Disc Damage Likelihood Scale, Heidelberg Retina Tomograph, and visual fields. Ophthalmology.

[22] Henderer JD, Liu C, Kesen M, Altangerel U, Bayer A, Steinmann WC, Spaeth GL (2003). Reliability of the disk damage likelihood scale. Am J Ophthalmol.

[23] Hornova J, Kuntz Navarro JB, Prasad A, Freitas DG, Nunes CM (2008). Correlation of Disc Damage Likelihood Scale, visual field, and Heidelberg Retina Tomograph II in patients with glaucoma. Eur J Ophthalmol.

[24] Spaeth GL, Henderer J, Liu C, Kesen M, Altangerel U, Bayer A, Katz LJ, Myers J, Rhee D, Steinmann W (2002). The disc damage likelihood scale: reproducibility of a new method of estimating the amount of optic nerve damage caused by glaucoma. Trans Am Ophthalmol Soc.

[25] Mantyjarvi M, Laitinen T (2001). Normal values for the Pelli-Robson contrast sensitivity test. J Cataract Refract Surg.

[26] Richman J, Zangalli C, Lu L, Wizov SS, Spaeth E, Spaeth GL, 1 (2014). The Spaeth/Richman contrast sensitivity test (SPARCS): design, reproducibility and ability to identify patients with glaucoma. Br J Ophthalmol.

[27] Warrian KJ, Lorenzana LL, Lankaranian D, Dugar J, Wizov SS, Spaeth GL (2010). The assessment of disability related to vision performance-based measure in diabetic retinopathy. Am J Ophthalmol.

[28] Mangione CM, Lee PP, Gutierrez PR, Spritzer K, Berry S, Hays RD (2001). Development of the 25-item National Eye Institute Visual Function Questionnaire. Arch Ophthalmol.

[29] Lee BL, Gutierrez P, Gordon M, Wilson MR, Cioffi GA, Ritch R, Sherwood M, Mangione CM (1998). The Glaucoma Symptom Scale. A brief index of glaucoma-specific symptoms. Arch Ophthalmol.

[30] Muthen B, Brown CH, Masyn K, Jo B, Khoo ST, Yang CC, Wang CP, Kellam SG, Carlin JB, Liao J (2002). General growth mixture modeling for randomized preventive interventions. Biostatistics (Oxford, England).

[31] Muthen B, Shedden K (1999). Finite mixture modeling with mixture outcomes using the EM algorithm. Biometrics.

[32] Elliott MR, Gallo JJ, Ten Have TR, Bogner HR, Katz IR (2005). Using a Bayesian latent growth curve model to identify trajectories of positive affect and negative events following myocardial infarction. Biostatistics (Oxford, England).

[33] Altangerel U, Spaeth GL, Steinmann WC (2006). Assessment of function related to vision (AFREV). Ophthalmic Epidemiol.

[34] Friedman DS, Freeman E, Munoz B, Jampel HD, West SK (2007). Glaucoma and mobility performance: the Salisbury Eye Evaluation Project. Ophthalmology.

[35] Haymes SA, Johnston AW, Heyes AD (2001). The development of the Melbourne low-vision ADL index: a measure of vision disability. Invest Ophthalmol Vis Sci.

[36] Haymes SA, LeBlanc RP, Nicolela MT, Chiasson LA, Chauhan BC (2008). Glaucoma and on-road driving performance. Invest Ophthalmol Vis Sci.

[37] Ramulu PY, West SK, Munoz B, Jampel HD, Friedman DS (2009). Glaucoma and reading speed: the Salisbury Eye Evaluation project. Arch Ophthalmol.

[38] Scott IU, Feuer WJ, Jacko JA (2002). Impact of visual function on computer task accuracy and reaction time in a cohort of patients with age-related macular degeneration. Am J Ophthalmol.

[39] Turano KA, Rubin GS, Quigley HA (1999). Mobility performance in glaucoma. Invest Ophthalmol Vis Sci.

[40] Guyatt GH (1993). Measurement of health-related quality of life in heart failure. J Am Coll Cardiol.

